# Retinal capillary and choriocapillaris assessment using a beam modifier optical coherence tomography angiography module to increase lateral optical resolution

**DOI:** 10.1371/journal.pone.0287783

**Published:** 2023-06-30

**Authors:** Sophie Bonnin, Sophie Kubach, Pierre Négrier, Warren Lewis, Luis de Sisternes, Aude Couturier, Ali Erginay, Marco Nassisi, Stephanie Magazzeni, Carlo Lavia, Ramin Tadayoni

**Affiliations:** 1 Ophthalmology Department, Hôpital Lariboisière, Assistance Publique des Hôpitaux de Paris, Université de Paris, Paris, France; 2 Fondation Adolphe de Rothschild, Paris, France; 3 R&D, Carl Zeiss Meditec Inc., Dublin, California, United States of America; 4 Department of Clinical Sciences and Community Health, University of Milan, Milan, Italy; 5 Ophthalmological Unit, Fondazione IRCCS Ca’ Granda Ospedale Maggiore Policlinico di Milano, Milan, Italy; 6 Surgical Department, Ophthalmology Service, Azienda Sanitaria Locale, Chieri, Italy; Northwestern University, Feinberg School of Medicine, UNITED STATES

## Abstract

**Purpose:**

To assess a new optical coherence tomography angiography (OCTA) technology and its contribution to retinal vascularization and choriocapillaris (CC) exploration.

**Methods:**

A new module, named “Beam expander” (BE), which increases the lateral resolution of OCTA, was used in combination with a prototype software in the PLEX® Elite 9000 Swept-Source OCT instrument (ZEISS, Dublin, CA). This prospective study involved 22 healthy subjects imaged with and without BE. Qualitative analysis of superficial capillary plexus (SCP), deep capillary complex (DCC) retinal and CC angiograms were performed. Perfusion density (PD), vessel density (VD), and foveal avascular zone (FAZ) measurements were also compared.

**Results:**

Qualitative analysis of single SCP and DCC retinal angiograms acquired with BE showed significantly better vessel sharpness (respectively, p = 0.0002, and p<0.0001), and greater peripheral image quality (p = 0.028 and p = 0.007) compared to standard OCTA images. Mean VD of whole retina single scans was significantly higher for BE angiograms compared to classic angiograms (28.16 ±1.29 mm^-1^ and 23.36 ±0.92 mm^-1^, respectively, p<0.0001). Repeatability of VD, PD and FAZ raw size were found to be similar between the two methods (intraclass correlation coefficient: 0.671, 0.604 and 0.994 with BE *versus* 0.764, 0.638 and 0.990 without BE). CC image quality was found to be significantly superior with BE, and flow deficits were more visible in all BE scans compared to standard scans.

**Conclusions:**

An increase in lateral resolution of the OCT beam resulted in higher quality of retinal and choriocapillaris OCTA images in healthy subjects. These results provide significant insights into the future OCTA imaging enhancements.

## Introduction

Optical coherence tomography angiography (OCTA) allows detecting non-invasively the retinal blood flow and obtaining its 3D representation, by analyzing differences within a repeatedly scanned transverse cross-sectional area of tissue [1]. Two of the current methods used for motion detection are amplitude decorrelation and phase variance. Amplitude decorrelation detects the difference in amplitude between the OCT B-scans while phase variance compares the changes in emitted light wave properties when it intercepts moving objects [1–3]. Both methods are based on the concept that a non-mobile tissue will remain identical while moving erythrocytes will cause changes in consecutive OCT scans. Both spectral-domain OCT (SD-OCT; wavelengths near 840 nm) and swept-source OCT (SS-OCT; wavelengths near 1,050 nm) use the same principles. The method used in PlexElite device for calculating the OCTA volume is OMAG [4], which uses eigen decomposition of a matrix composed of repeated complex OCT B-scans to isolate the varying components, assumed to be due to moving blood cells, from the signal from static tissue. This phase sensitive method is sensitive to movements that may not be detectable with phase insensitive OCTA algorithms.

The clinical significance of OCTA continues to mature as the use of the technology grows.

The management of retinal diseases such as diabetic retinopathy or age-related macular degeneration is facilitated by the use of OCTA [5, 6]. But there are some limitations, including the fact that the size of the blood vessels on OCTA is overestimated compared to histology, due to a widening of the apparent diameter of retinal vessels. This effect is evident when comparing OCTA and adaptive optics scanning light ophthalmoscope fluorescein angiography scans [7]. Image averaging, that has been introduced more recently, enhances image quality by improving the signal-to-noise ratio [8, 9], resulting in an enhanced visualization of the retina and choriocapillaris in the OCTA scans.

Engineers continue to develop solutions to improve OCTA scan quality and facilitate the diagnostic process: the “Beam Expander” (BE) module was developed to be installed on the PlexElite machine. It consists of a pair of lenses that magnify the beam size at the pupil plane, hence the name of “Beam expander”, while increasing the lateral optical resolution of the beam at the retina and the choroid.

The aim of this study was to assess the qualitative and quantitative effects of an increase in lateral resolution of the OCT beam on the retinal and choriocapillaris en face images from both single and average OCTA scans in a cohort of healthy subjects.

## Material and methods

The study was conducted in compliance with the tenets of the Declaration of Helsinki. The collection of all study-related data was approved by the Institutional Review Board (CEERB d’Ile de France, Paris, France). Subjects provided their written informed consent.

This prospective study was conducted in Lariboisière Hospital, Paris, France, between July 1, 2019 and January 30, 2020. Twenty-two healthy volunteers without ocular disease and with normal (i.e., 20/20) best-corrected visual acuity were included. Ocular fundus examination was normal in all eyes, and their refraction ranged between −4 and +2 diopters. All eyes were imaged with SS-OCTA using the PLEX® Elite 9000 Swept-Source OCTA device (Carl Zeiss Meditec Inc., Dublin, CA, USA). Scans were analyzed using a modified 2.1 prototype software version of the instrument.

For each subject, the right eye was included in the study.

### Optical coherence tomography angiography (OCTA) device and beam expander module

Study eyes were imaged using the SS-OCTA PlexElite 9000 device that uses a swept-source, tunable laser operating at a center wavelength of 1,060 nm and at the dual-speed of 100,000 A-scans per second with an A-scan depth of 3 mm or 6 mm in tissue, an axial optical resolution of about 6.3 μm and a transverse resolution of about 25 μm at the retina.

A prototype software was used on the OCT instrument in combination with the BE module in the OCT path: it included required changes to handle the module and control new functionalities offered by it. Without the BE module, the beam at the pupil plane has a 0.86 mm diameter, which translates to a beam diameter of about 25 μm at the retina. In order to verify our model of the system, the beam was measured at the pupil plane using a Charge-Coupled Device. Agreement within about 1% was obtained between the measured beam diameter and that from the model. The measured profile was used to determine the beam diameter, and this was used via Fourier transform to estimate the spot size at the retina, assuming a diffraction limited optical system. This result was found to be in close agreement with the Zemax optical model. The BE module, consisting of a matched negative and positive lens in the configuration of a Galilean telescope with magnification 2.2x, is placed in the optical system in advance of the scanning galvanometer, which limits the beam size, acting as an aperture in the system. The introduction of the BE module results in an increased beam size at the ocular pupil plane. The BE module magnifies the beam by a 2.2 factor resulting in a beam diameter of 1.9 mm at the pupil plane. This beam size, in combination with the effects of the effective aperture at the galvanometers, produces a beam diameter estimated to be about 14 μm at the retinal plane. As a consequence, with the beam expander in place, the depth of focus at the retina is reduced. The Rayleigh range of the OCT beam, which corresponds to the tolerance on the position of the beam waist at the retina, is about 105 μm in the system with the BE in place. This may be compared to about 510 μm for the system without the BE.

For this study, each eye was therefore imaged with 3×3-mm (standard scan) and 2.25x2.25-mm (BE scan) OCTA scans. Acquisitions for both the standard and BE scan were repeated three times per eye for the purpose of analyzing image repeatibility. Of note, data analysis (qualitative and quantitative) was performed on single scans, as regularly done in clinical practice. Both scans consisted of 300 B-scans of 300 A-scans. All scans were captured using the FastTrack eye motion correction software (Carl Zeiss Meditec, Inc, USA). The scan pattern for the configuration with the BE consists of 300x300 samples over a 2.25x2.25 mm field of view i.e. 7.5 μm sampling resolution. The scan pattern for the configuration without the beam expander consists of 300x300 samples over a 3 mm x 3mm field of view or a 10 μm sampling resolution. The quality of all OCTA scans was verified in the quality check screen after completion of the scan. Scans were not included in this analysis if a signal strength lower than 7, significant motion artifacts or evidence of defocus or blur were present on more than 10% of the image.

Exported OCTA volumes were uploaded to the ARI Network platform (https://arinetworkhub.com), a cloud-based collaborative and processing solution provided by Zeiss and available to clinical and research institutions who are part of the A R I network. The three repeated standard and BE scans were processed off-line on Matlab (The MathWorks, Inc., Natick, Massachusetts, USA) using an algorithm (created by Carl Zeiss Meditec) that registers the cube scans relative to each other and then averages them. The layer segmentation algorithm is a proprietary algorithm that outlines the location of the retinal layers within the OCT data. This algorithm is provided by the device manufacturer and part of the review software available in the device. The algorithm selects a reference retinal slab and calculates the transformation vectors to correctly register the slabs from the other repeated scans relative to the reference slab. The transformation vectors are applied along all A-scan vectors to produce registered angio cubes. The registered cubes are then averaged.

### Qualitative analysis of OCTA images

The BE and standard OCTA scans were aligned using ImageJ software (National Institutes of Health, Bethesda, MD): the large vessels in the superficial capillary plexus (SCP) scans were used as a reference for the alignment of all the corresponding scans (i.e. the deep capillary complex [DCC], and choriocapillaris scans). The standard scans were cropped to obtain the same size as that of the BE scans, so that they were undistinguishable for grading purposes of the qualitative criteria.

**Fig 1:**
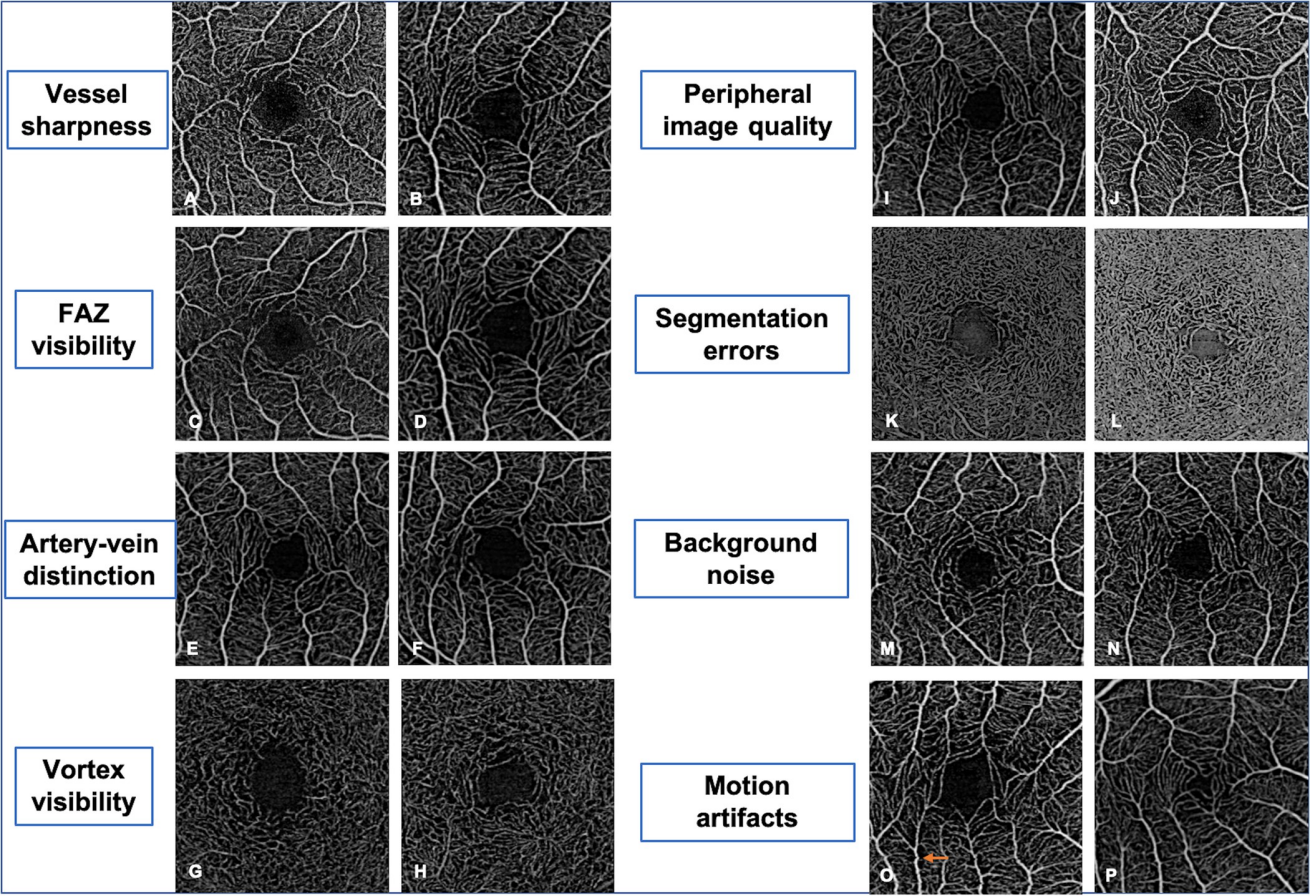
Retinal image quality criteria (3x3-mm angiograms) in the superficial capillary plexus (SCP) and deep capillary complex (DCC). A.B. Vessel sharpness, SCP (A: 0 = poor; B: 2 = good); C.D. FAZ visibility, SCP (C: 0 = poor; D: 2 = good); E.F. Artery-vein distinction, SCP (E: 0 = poor; F: 2 = good); G.H. Vortex visibility, DCC (G: poor; H: 2 = good); I.J. Peripheral image quality, SCP (I: poor; J: 2 = good); K.L. Segmentation errors, DCC (K: poor; L: 2 = good); M.N. Background noise, SCP (M: poor; N: 2 = good); O.P. Motion artifacts, SCP (G: poor; H: 2 = good). The orange arrow on image O is showing the discontinuity of vessel course (motion artifact).

Eight quality criteria were assessed for the standard and BE scans (in both single and average images), for the analysis of the SCP and DCC scans (Fig 1). Comparisons were made between single standard *versus* BE scans, and between average standard *versus* BE scans.

The eight SCP and DCC image quality criteria were: 1) vessel sharpness (i.e., the presence of a clear distinction between the flow and no-flow areas detected by the machine within the whole scan); 2) foveal avascular zone (FAZ) visibility (i.e., the ability to clearly see and follow the course of capillaries surrounding the FAZ); 3) artery-vein distinction (i.e., the visibility of larger avascular areas around pre-capillary arterioles compared to post-capillary venules within the whole scan); 4) vortex visibility (i.e., the ability to identify capillaries with a specific vortex pattern, as previously described [5]); 5) comparison of the scan quality in the periphery *versus* center (i.e., the uniformity of image quality, including vessel sharpness); 6) presence of segmentation errors (i.e., the presence of any segmentation error <10% of the total scan area; in presence of segmentation errors, a grade 0 was assigned); 7) background noise (i.e., the presence of a false flow signal in no-flow areas, particularly visible within the FAZ); 8) presence of motion artifacts (i.e., the presence of any misalignment visible as a discontinuity in the vessel course <10% of the total scan area) (Fig 1).

Criteria 1, 5, 6, 7 and 8 were assessed on both the SCP and DCC slabs. Criterion 2 was assessed on the full-retina slab. Criterion 3 was assessed on the SCP slab. Criterion 4 was assessed on the DCC slab.

The grading process was set up by three OCT masked experts (SB, CL, MN) and reference images were used for comparisons for the entire grading process. Two experts graded the retinal criteria (SB, CL) and two experts graded the choriocapillaris criteria (CL, MN). Data from qualitative analysis obtained from the two graders were averaged to perform statistical analysis.

All criteria except the vortex visibility were graded from 0 to 2 (0: poor; 1: medium; 2: good).

Vortex visibility was compared between the 2 acquisition methods and was graded 1, 0 or -1 (-1 corresponding to a better quality for the BE image; 0 corresponding to no difference, and 1 corresponding to better quality in the standard scan).

**Fig 2:**
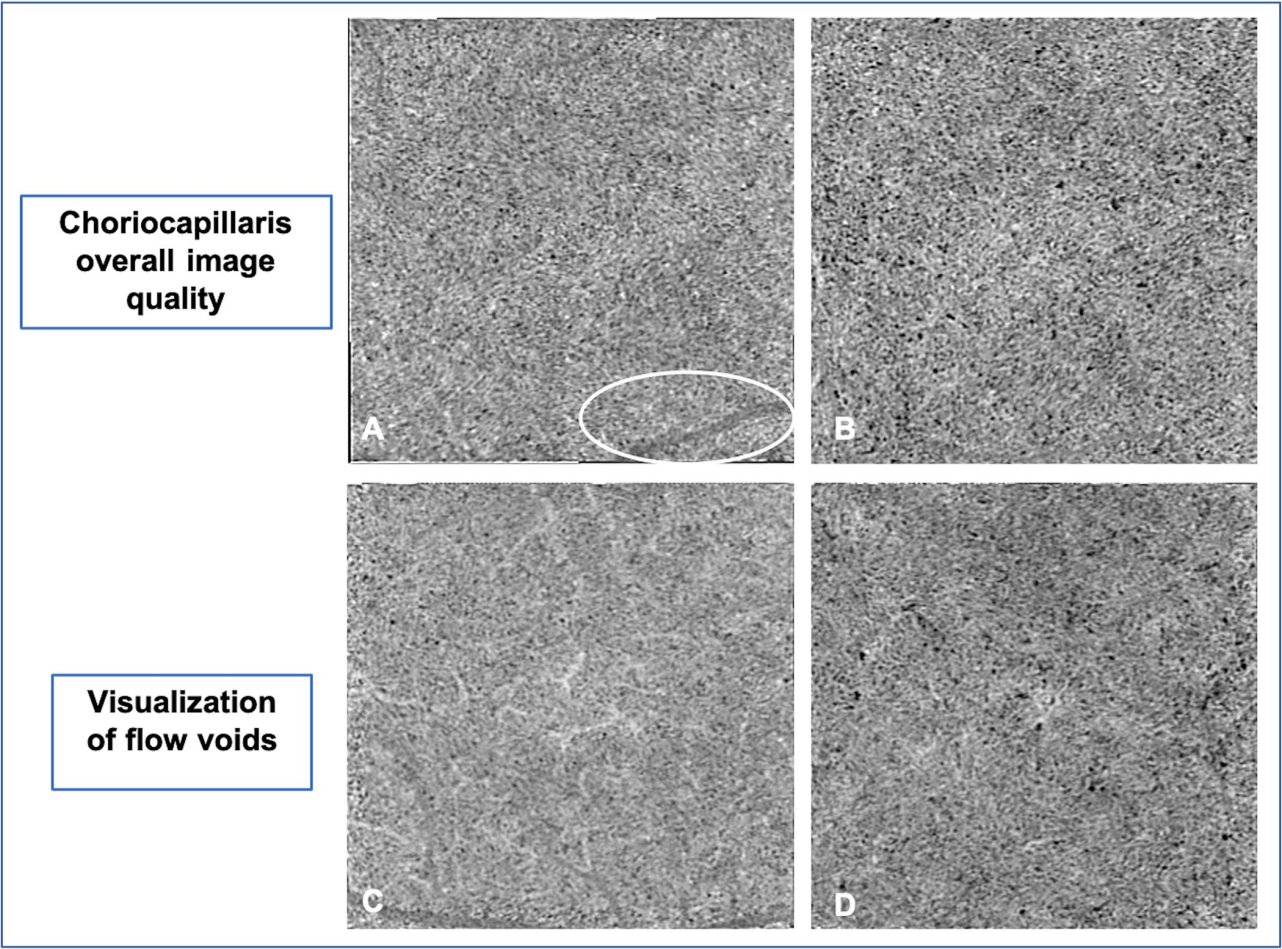
Choriocapillaris image quality assessment (3x3-mm angiograms). The overall image quality seems greater in B compared to A with higher uniformity and capillary sharpness, and fewer artifacts (for example, one projection artifact is showed by the white circle) (A: medium overall image quality; B: good overall image quality). The visualization of flow voids is enhanced in D compared to C with a better contrast in flow and no-flow areas (C: medium visualization of flow voids; D: good visualization of flow voids).

For the choriocapillaris analysis, single scans were compared. The high-density characteristics of the choriocapillaris in comparison with other slab makes the choriocapillaris slab more susceptible to registration error and more challenging to obtain good quality averaged image. Two quality criteria were assessed: 1) overall image quality (i.e., an overall assessment of capillary sharpness, image quality and uniformity), and 2) visualization of flow voids (i.e., the ability to clearly delineate flow voids, based on the contrast between the flow and no-flow areas). Both criteria were graded with a score from 0 to 2 (0: poor; 1: medium; 2: good) (Fig 2).

### Quantitative analysis of OCTA images

Standard and BE single scans were processed using the ‘Density Quantification v0.3.5’ algorithm available on the ARI network portal that uses a multilayer segmentation and calculates vessel metrics for the SCP, the DCC and the whole retina. Quantitative comparisons between standard and BE scans involved the same area (a 1-2-mm diameter annulus centered on the fovea).

**Fig 3:**
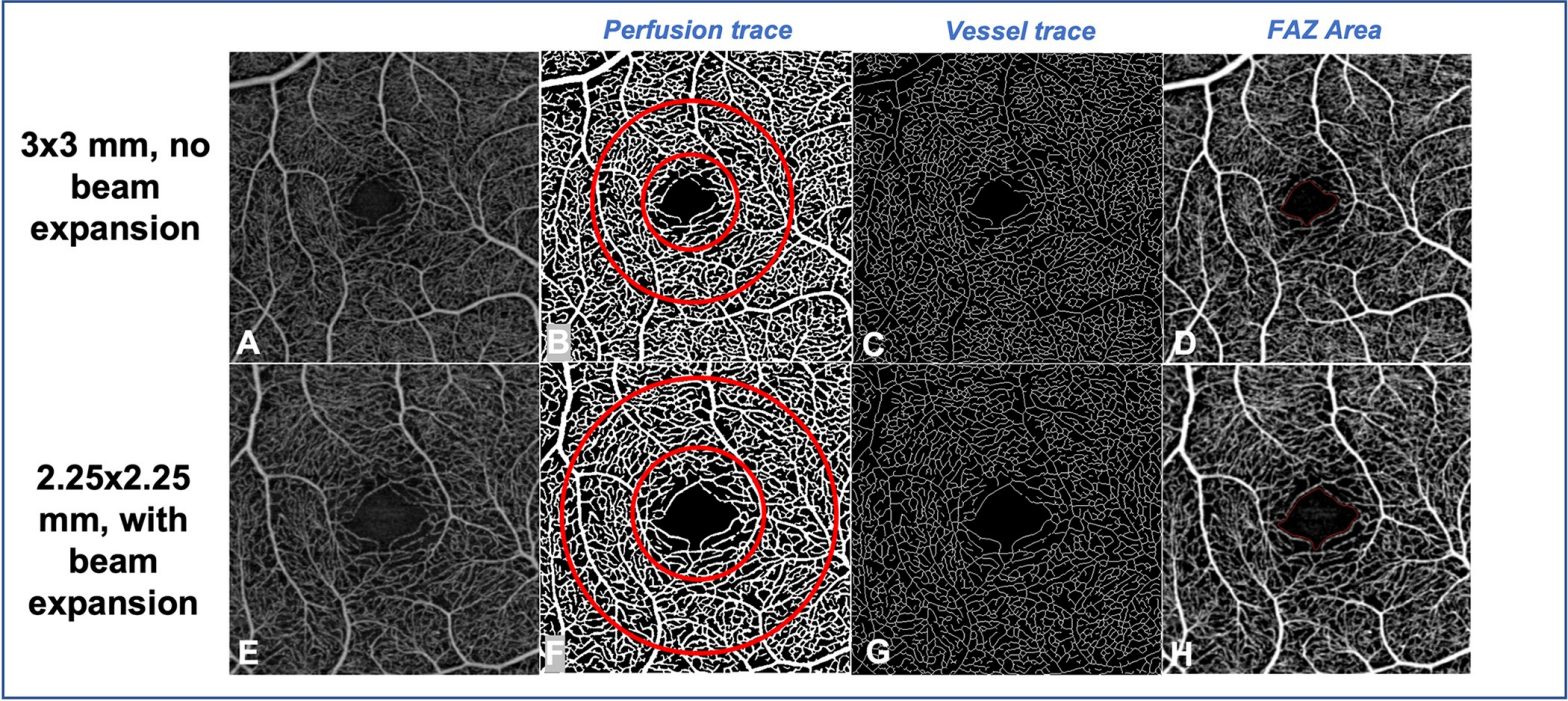
Comparison of quantitative measurements in 3x3-mm angiograms (A, B, C, D) and 2.25x2.25-mm angiograms (D, F, G, H). A and E: Superficial capillary plexus angiograms. B and F: Perfusion trace allowing measuring perfusion density, and showing the inner ring used to compare the measurements. This inner ring is delimited by a 2-mm diameter circle, and a 1-mm diameter circle (in red). C and G: Vessel trace allowing measuring perfusion density. D and H: The foveal avascular zone is delineated in red.

Perfusion density (PD) is defined as the area of detected flow per unit area, given in %. PD is calculated by thresholding the *en*-face OCTA images, resulting in a binary image where each pixel corresponds to a perfused or non-perfused area (Fig 3). The vessels are first enhanced using a Hessian filter based on Franji method [10] and then thresholding is performed based on the noise level. The noise is determined from the scan volume by identifying areas with no structure present and measuring the average levels there. This value is then used to threshold the en-face projections.

Vessel density (VD) is defined as the total vessel length per unit area in a measurement area. It is measured in units of inverse millimeters (mm^-1^). The binarized images corresponding to the PD are skeletonized to represent the vessels by their centerlines (traces with 1-pixel width) (Fig 3). The algorithm of skeletonization is a proprietary algorithm that 1. applies a Frangi vesselness filter to the angio en face, 2. thresholds the image, 3. performs a number of binary operations to remove features unlikely to correspond to actual vascular structure to obtain the perfusion binary image, then skeletonizes this perfusion image using an erosion technique to reduce the vessel widths to 1 pixel. The main difference between the VD and the PD is that all vessels are considered equal regardless of the size in the VD while in the PD, larger vessels influence the measurement more than smaller capillaries and therefore, can mask the loss of individual capillaries. The PD and the VD are unique to the PlexElite software and are commonly referred, respectively, as vessel density and vessel length density in the literature.

The FAZ was assessed by measuring its size (mm^2^) and circularity (relative to a circle of the same size, dimensionless). Circularity is defined to be (4*pi*FAZarea/FAZperimeter^2). In the present study, quantitative parameters were analyzed for the SCP and whole retina slabs.

### Statistics

The comparisons of both qualitative and quantitative criteria were performed using the Wilcoxon signed-rank test. The repeatability of the quantitative measurements was assessed using the intraclass correlation coefficient (ICC) with its 95% confidence interval, based on an absolute agreement, two-way mixed-effect model. P values <0.05 were considered significant. All statistical analyzes were performed using GraphPad Prism 9.0 (GraphPad Software, Inc., San Diego, CA).

## Results

26 eyes of healthy subjects were imaged with the BE module; 4 eyes were excluded due to the presence of image artifacts, preventing the analysis of the images. Finally, a total of 22 eyes of 22 healthy subjects (13 women and 9 men) were included. The mean ± standard deviation age of the subjects was 31.5 ± 5.6 years (23–44 years).

### Qualitative analysis

**Fig 4:**
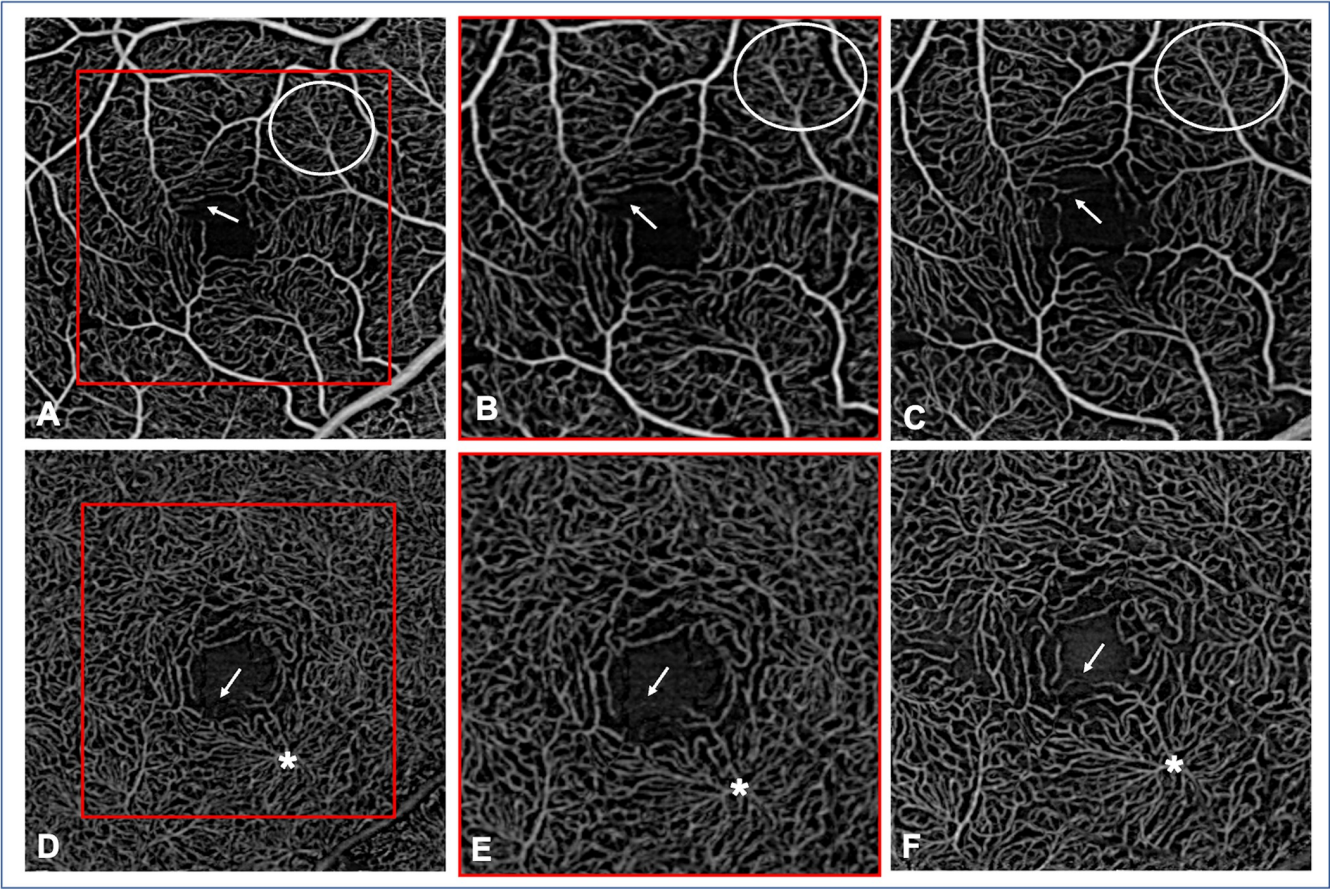
Comparison between 3x3-mm standard angiograms (A, D), the same 3x3 mm angiograms cropped to a 2.25x2.25 mm field of view (B, E) and 2.25x2.25-mm angiograms (C, F) acquired with the Beam Expander (BE). A, B and C: Superficial capillary plexus; D, E and F: Deep capillary complex (DCC). The vessels are more continuous (as circled on the images A, B and C) in the BE scans, resulting in a higher uniformity of image quality. Some vessels are more visible (white arrow), contributing to improve the visualization of the foveal avascular zone. The radial organization of the vortex in the DCC is better defined in F than in E (white asterisk).

The SCP and DCC scans acquired with the BE showed thinner capillaries, a longer intercapillary distance (Fig 4A–4C), enhanced vortex visualization (Fig 4D–4F) and FAZ visibility compared to the standard scans.

The qualitative analysis of the single scans showed significantly higher vessel sharpness scores (in both the SCP and the DCC; p = 0.0002 and p <0.0001, respectively) and peripheral image quality scores (in both the SCP and the DCC; p = 0.002 and p <0.012, respectively) for the BE scans compared to the standard scans (Table 1). The vortexes were more easily identified in 68% of BE scans, while in the remaining 32%, their visualization was comparable between the BE and standard scans. Regarding the other image quality criteria (Table 1), no significant difference was found between both acquisition methods.

**Table 1:** Comparison of the qualitative criteria used for the single scans acquired with and without the beam expander (BE) module.

	Single scans					
	SCP			DCC		
Scores	3x3 mm	2.25x2.25 mm (BE)	*P value*	3x3 mm	2.25x2.25 mm (BE)	*P value*
**Vessel sharpness**	1.05 (±0.38)	1.46 (±0.43)	**0.0002**	0.95 (±0.34)	1.48 (±0.36)	**<0.0001**
**Mean (**±**SD)**						
**FAZ delineation**	1.23 (±0.43)	1.43 (±0.42)	0.18	N/A	N/A	
**Mean (**±SD)*						
**Artery-Vein distinction**	1.55 (±0.34)	1.5 (±0.38)	0.77	N/A	N/A	
**Mean (**±**SD)**						
**Periphery/center**	1.27 (±0.46)	1.57 (±0.42)	**0.002**	1.21 (±0.43)	1.5 (±0.38)	**0.012**
**Mean (**±**SD)**						
**Segmentation errors**	1.68 (±0.42)	1.55 (±0.49)	0.27	1.43 (±0.42)	1.18 (±0.48)	0.65
**Mean (**±**SD)**						
**Background noise**	1.46 (±0.43)	1.32 (±0.36)	0.33	1.32 (±0.36)	1.32 (±0.33)	>0.9999
**Mean (**±**SD)**						
**Motion artifacts**	1.23 (±0.48)	1.16 (±0.39)	0.68	1.3 (±0.45)	1.3 (±0.37)	>0.9999
**Mean (**±**SD)**						
**Vortex visibility**	N/A	N/A		-0.64 (±0.47)		
**Mean (**±**SD)**						

SCP: Superficial Capillary Plexus; DCC: Deep Capillary Complex; FAZ: foveal avascular zone; SD: Standard Deviation; N/A: non applicable.

*: FAZ evaluation was performed on the full retina slab.

The qualitative analysis of the average scans showed significantly higher vessel sharpness scores (in both the SCP and the DCC; p = 0.0001 and p <0.0001, respectively), peripheral image quality scores (in both the SCP and the DCC; p = 0.028 and p = 0.007, respectively), FAZ delineation scores (p = 0.002) and artery-vein distinction scores (p = 0.03) for the BE scans compared to the standard scans. No significant difference was found between both acquisition methods, regarding the assessment of segmentation errors and motion artifacts. Vortex visibility was enhanced in all scans acquired with the BE (Table 2).

**Table 2:** Comparison of the qualitative criteria used for the average scans acquired with and without the beam expander (BE) module.

	Average scans						
	SCP			DCC			
Scores	3x3 mm	2.25x2.25 mm (BE)	*P value*	3x3 mm	2.25x2.25 mm (BE)		*P value*
**Vessel sharpness**	1.3 (±0.43)	1.86 (±0.38)	**0.0001**	1.23 (±0.40)	1.91 (±0.33)		**<0.0001**
**Mean (**±**SD)**							
**FAZ delineation**	1.5 (±0.44)	1.86 (±0.32)	**0.002**	N/A	N/A		
**Mean (**±SD)*							
**Artery-Vein distinction**	1.66 (±0.47)	1.96 (±0.2132)	**0.031**	N/A	N/A		
**Mean (**±**SD)**							
**Periphery/center**	1.48 (±0.36)	1.75 (±0.51)	**0.028**	1.46 (±0.41)	1.8 (±0.37)		**0.007**
**Mean (**±**SD)**							
**Segmentation errors**	1.93 (±0.18)	1.86 (±0.47)	0.81	1.46 (±0.49)	1.39 (±0.46)		0.27
**Mean (**±**SD)**							
**Background noise**	1.89 (±0.34)	1.96 (±0.11)	0.5	1.66 (±0.42)	1.91 (±0.2)		**0.028**
**Mean (**±**SD)**							
**Motion artifacts**	1.8 (±0.37)	1.75 (±0.34)	0.75	1.8 (±0.3671)	1.84 (±0.28)		0.79
**Mean (**±**SD)**							
**Vortex visibility**	N/A	N/A		-0.77 (±0.48)			
**Mean (**±**SD)**							

Abbreviations. SCP: Superficial Capillary Plexus; DCC: Deep Capillary Complex; FAZ: foveal avascular zone; SD: Standard deviation; N/A: non applicable.

*: FAZ evaluation was performed on the full retina slab.

**Fig 5:**
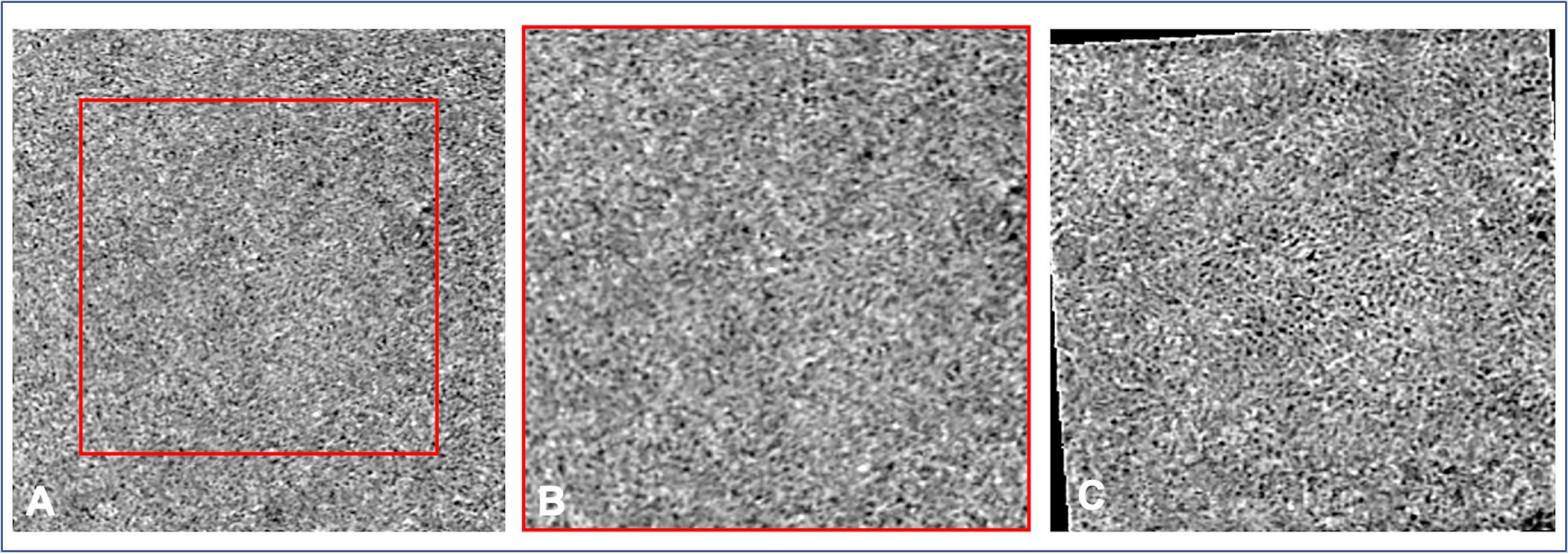
Comparison of choriocapillaris images from the same healthy subject. A. 3x3-mm standard angiogram. B. Same 3x3-mm angiogram cropped to match the scan area of the corresponding 2.25x2.25-mm angiogram. C. 2.25x2.25-mm angiogram acquired with the Beam Expander and registered using the superficial slab of the to the 3x3 mm angiogram for comparison. The flow voids are more distinct in C and the capillary structures are better defined.

The single choriocapillaris slabs acquired with the BE module showed significantly higher overall image quality scores (1.43 ± 0.49 *versus* 1.07 ± 0.32, p = 0.014) and flow void visualization scores (1.84 ± 0.32 *versus* 1.14 ± 0.23, p <0.0001) compared to the standard scans (Fig 5).

### Quantitative analysis

The mean VD in the whole retina, in the 1-2-mm diameter annulus, was significantly higher in the BE scans compared to the standard scans (28.16 ±1.29 mm^-1^ and 23.36 ±0.92 mm^-1^, respectively, p<0.0001) and the mean PD in the whole retina in the same area was significantly lower in the BE scans compared to the standard scans (0.43 ± 0.02 for the BE scans *versus* 0.44 ± 0.01 for the standard scans, p = 0.003).

The mean FAZ circularity was significantly lower in the BE scans compared to the standard scans (0.67 ± 0.09 mm^-1^ *versus* 0.7 ± 0.07 mm^-1^, p = 0.03), whereas the mean FAZ area was not significantly different between both acquisition methods (Table 3). Of note, automatic segmentation failed in two cases (one BE and one standard scan) and data from the second of the three scans acquired was used.

**Table 3:** Comparison of the quantitative criteria used for the single whole retina scans acquired with and without the beam expander (BE) module.

	3x3 mm	2.25x2.25 mm (BE)	*P value*
**Vessel density (mm** ^**-1**^ **)**	23.36 (±0.92)	28.16 (±1.29)	**<0.0001**
**Mean (**±**SD)**			
**Perfusion density (%)**	0.44 (±0.01)	0.43 (±0.02)	**0.0031**
**Mean (**±**SD)**			
**FAZ circularity (mm** ^**-1**^ **)**	0.7(±0.07)	0.67 (±0.09)	**0.03**
**Mean (**±**SD)**			
**FAZ raw size (mm** ^**2**^ **)**	0.18 (±0.07)	0.18 (±0.07)	0.41
**Mean (**±**SD)**			

Abbreviations. FAZ: foveal avascular zone; SD: Standard deviation

VD, PD and FAZ raw size measurement repeatability was slightly lower in the BE scans compared to the standard scans (ICC: 0.671 (0.457–0.833), 0.604 (0.367–0.764) and 0.994 (0.987–0.997) for BE scans *versus* 0.764 (0.589–0.884), 0.638 (0.409–0.814) and 0.990 (0.980–0.996) for the standard scans).

## Discussion

This analysis showed the benefit of an increased lateral resolution and sampling density on the quality of the retinal and choriocapillaris OCT angiograms in a cohort of healthy subjects. The qualitative analysis of the retinal OCTA single scans showed enhanced vessel sharpness and image quality uniformity in the BE scans compared to the standard scans. The enhanced visibility and sharpness of the retinal capillaries, arterioles and venules could more efficiently reveal subtle and preclinical pathological findings that could be difficult to detect with a quantitative assessment if they are localized. Moreover, an increased lateral resolution and sampling density in the deeper slabs could improve the visibility of nascent retinal or choroidal vessels in macular neovascular diseases.

In the choriocapillaris slabs, reliable images of the choriocapillaris lobular pattern as reported in histological studies are usually difficult to obtain, partly because of a problem of image resolution associated with the current OCT devices, resulting in an overestimation of the caliber of retinal and choriocapillaris vessels [11, 12]. The choriocapillaris flow signal is weak as it is attenuated by neuronal and pigmented (i.e., retinal pigment epithelium) structures. It remains challenging to image the choriocapillaris *in vivo* [13, 14]. Recently, SS-OCTA has been shown to enhance choriocapillaris images, and the quantification of flow voids has become a key quantification criteria [15].

The greater tissue penetration of SS-OCTA combined with the increased lateral and sampling resolution of the BE scans allowed obtaining spectacular images of the choriocapillaris meshwork and flow voids (Fig 5). Qualitatively, the flow voids were more clearly visible in the BE scans than in the standard scans.

The qualitative analysis performed in this study included both “anatomical” (e.g. artery-vein distinction or vortex visualization) and “structural” (e.g. segmentation errors, vessel sharpness) criteria, previously used in other studies [16–18]. The qualitative analysis of OCT and OCTA images is usually based on software-derived image quality scores (e.g. signal strength index), based on pixel intensity, without considering other aspects that could significantly influence the understanding of the acquired data, such as vortex visibility or segmentation errors. Shahlaee et al. [16] have suggested criteria to be used to qualitatively assess the macular VD on OCTA, and recommended to exclude scans with motion or blink artifacts, signs of media opacities and incorrect vascular network segmentation. More recently, Fenner et al. [17] have assessed several image quality parameters, including the automatically generated “TopQ score”, and reported that the repeatability of retinal capillary plexus density measurements was affected by the presence of motion artifacts in the SCP and by the low visibility of fine vessels, the presence of motion artifacts and B-scan quality in the DCC. The Peak signal-to-noise ratio is also an interesting index for quantitative comparison of the image quality [19].

Our assessment of artery-vein distinction in the SCP and capillary vortex visibility in the DCC was based on OCTA anatomical findings [5, 18]. The capillary-free zone along the retinal arteries allowed distinguishing arteries and veins in the SCP [20].

Our qualitative analysis showed that an increased lateral resolution and sampling resolution affected more significantly anatomical rather than structural criteria. This finding could be explained by the sample examined, composed of healthy subjects with no media opacity and good fixation.

**Fig 6:**
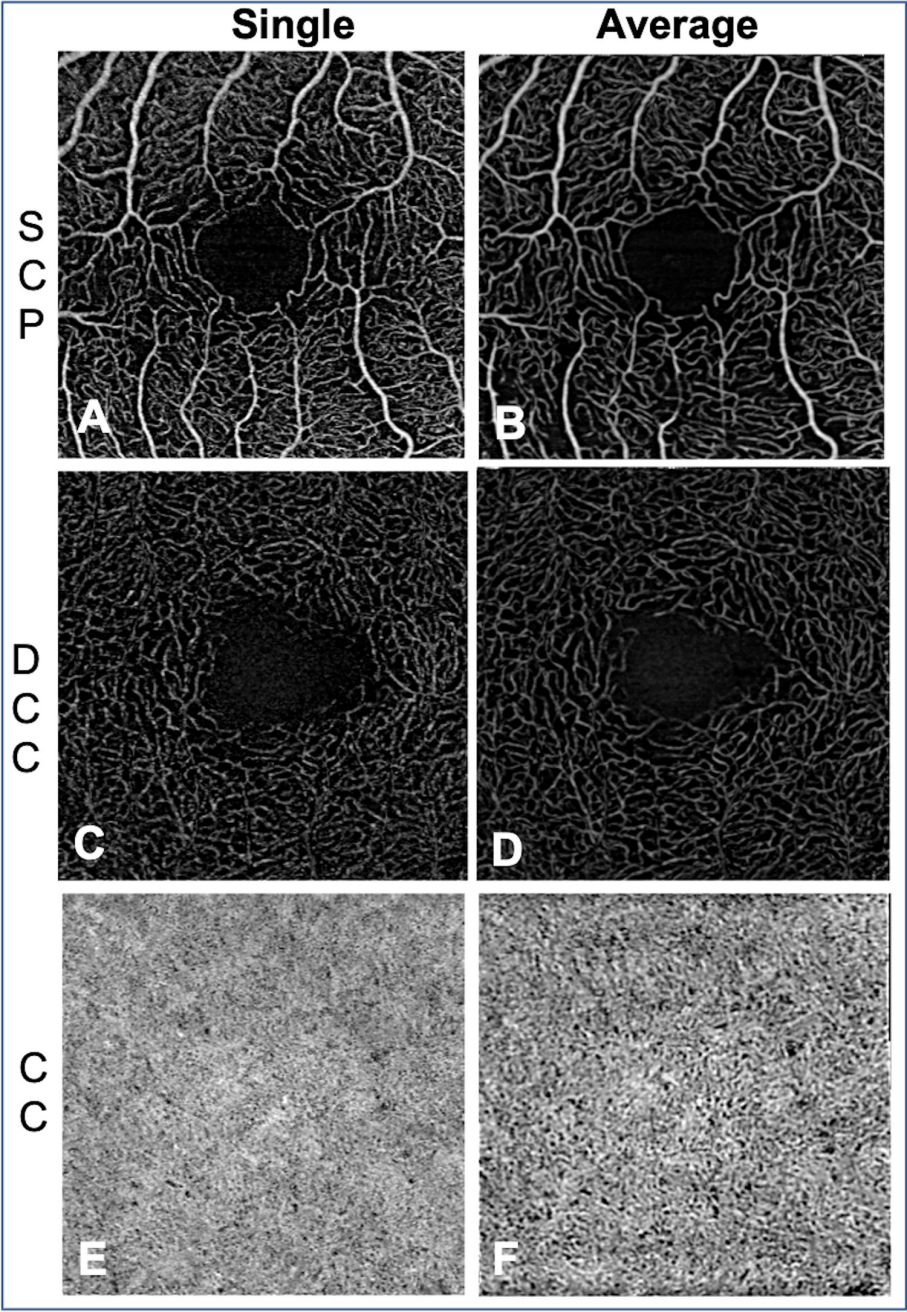
Comparison between single (A, C, E) and average (B, D, E) 2.25x2.25-mm angiograms (performed with Beam Expander). A and B: Superficial capillary plexus (SCP); C and D: Deep capillary complex; E and F: Choriocapillaris.

Image averaging further improved the qualitative assessment of retinal slabs. Vessel sharpness, image quality in the periphery *versus* center, FAZ visibility, artery-vein distinction and vortex visibility were enhanced in the averaged scans compared to the single scans (Fig 6). And averaged BE scans in the SCP and DCP presented greater vessel sharpness and peripheral versus central image quality compared to averaged 3x3 mm standard scans (Table 2).

The comparison between average 3x3 mm and single BE scans showed significant difference in the visualization of the DCP while no significant difference was observed for the remaining anatomical criteria (Table 4).

**Table 4:** Qualitative criteria: comparison between single BE and averaged 3x3 mm scans.

	Single 2.25x2.25 scans vs Average 3x3 scans					
	SCP			DCC		
**Scores**	**Average 3x3 mm**	**Single 2.25x2.25 mm (BE)**	*P value*	**Average 3x3 mm**	**Single 2.25x2.25 mm (BE)**	*P value*
**Vessel sharpness**	1.3 (±0.43)	1.46 (±0.43)	0.14	1.23 (±0.40)	1.48 (±0.36)	**0.02**
**Mean (**±**SD)**						
**FAZ delineation**	1.5 (±0.44)	1.43 (±0.42)	0.67	N/A	N/A	
**Mean (**±**SD)**						
**Artery-Vein distinction**	1.66 (±0.47)	1.5 (±0.38)	0.25	N/A	N/A	
**Mean (**±**SD)**						
**Periphery/center**	1.48 (±0.36)	1.57 (±0.42)	0.42	1.46 (±0.41)	1.5 (±0.38)	0.71
**Mean (**±**SD)**						
**Segmentation errors**	1.93 (±0.18)	1.55 (±0.49)	**0.0049**	1.46 (±0.49)	1.18 (±0.48)	**0.06**
**Mean (**±**SD)**						
**Background noise**	1.89 (±0.34)	1.32 (±0.36)	**0.0002**	1.66 (±0.42)	1.32 (±0.33)	**0.017**
**Mean (**±**SD)**						
**Motion artifacts**	1.8 (±0.37)	1.16 (±0.39)	**<0.0001**	1.84 (±0.28)	1.3 (±0.37)	**0.0009**
**Mean (**±**SD)**						

Abbreviations. SCP: Superficial Capillary Plexus; DCC: Deep Capillary Complex; FAZ: foveal avascular zone; SD: Standard deviation; N/A: non applicable

The benefit of averaging OCTA images has been previously shown in the assessment of anatomical structures [21, 22]. Previous studies have shown that multiple image averaging improves en face OCTA image quality by reducing the background noise and enhancing the image contrast [9, 19, 23]. However, image averaging is not always practical due to prolonged acquisition times, the need for image post-processing and the risk of losing some image details as previously reported. In our study, the qualitative and quantitative differences observed between the standard and BE scans persisted after image averaging.

Regarding the quantitative analysis, our results showed a significantly higher mean VD in the BE scans compared to the standard scans. This could be explained by the enhanced lateral and sampling resolution of the BE scans, that presented lower number of discontinuous capillaries and missed capillary segments specially if they are narrow, which could be helpful in the evaluation of healthy and diseased eyes.

The significant lower FAZ circularity found in the BE scans is likely the result of improved vessel continuity from the increased lateral and sampling resolution. We found also that the FAZ circularity was significantly lower in the BE scans by using only the superficial slabs (p = 0.005) (Table 5). FAZ measurement is critical in clinical practice since previous studies have shown that the FAZ allows predicting DR progression [24].

**Table 5:** Comparison of the quantitative criteria used for the single superficial scans acquired with and without the Beam Expander (BE) module.

	3x3 mm	2.25x2.25 mm (BE)	*P value*
**Vessel density (mm** ^**-1**^ **)**	21.5 (±1.18)	25.61 (±1.2)	**<0.0001**	
**Mean (**±**SD)**				
**Perfusion density (%)**	0.41 (±0.02)	0.40 (±0.02)	0.07	
**Mean (**±**SD)**				
**FAZ circularity (mm** ^**-1**^ **)**	0.71(±0.075)	0.68 (±0.08)	**0.005**	
**Mean (**±**SD)**				
**FAZ raw size (mm** ^**2**^ **)**	0.18 (±0.07)	0.17 (±0.07)	0.97	
**Mean (**±**SD)**				

Abbreviations. FAZ: foveal avascular zone; SD: Standard deviation

The main limitation of our study is the small size of the sample composed of young healthy subjects. Future studies in larger cohorts, focused on retinal and choroidal diseases are needed to confirm the usefulness of the BE. The increased optical resolution provided by the BE is counterbalanced by a reduced depth of focus, resulting in a higher sensitivity to defocus. In these young subjects with good fixation, this reduced depth of focus was not an issue, but the enhanced quality has to be confirmed in macular diseases. The excellent image quality of the scans obtained in this study of healthy subjects allowed the regularity of the retinal layers, and insured against significant projection artifacts. But we paid attention to retinal segmentation by scrolling all the B-scans and we carefully examined all the en-face scans to highlight any suspected projection artifact. Another limitation of this technology is the absence of wider-field images available, at this time. However, this pilot study aimed to investigate the repeatability and the potential usefulness of the BE and it was necessary to perform the examinations under the best conditions of fixation and media transparency before assessing diseased eyes. Secondly, a quantitative analysis of the choriocapillaris slabs was not performed. Due to the lack of consensus on choriocapillaris flow void quantification [12], we preferred not to perform any image post-processing and to limit our analysis to a qualitative assessment. Even considering its subjectivity, BE images seemed closer to histological images of the choriocapillaris than standard images.

In conclusion, this study showed that by increasing the lateral and sampling resolution, the OCTA image quality could be improved and that, as a consequence, a more reliable flow quantification could be achieved. Further analyzes of eyes with retinal and choroidal disorders are needed to assess BE usefulness in macular diseases. Moreover, if the BE could be used in larger scans, it could provide useful insights into the assessment of the perifoveal retinal vascularization in retinal vascular diseases.

## Supporting information

S1 Data (XLSX)
